# 

**Published:** 1894-03

**Authors:** 


### THE BRISTOL

### Medico-Chirurgical Journal.

A JOURNAL OF THE MEDICAL SCIENCES FOR THE WEST OF ENGLAND AND SOUTH WALES

*PUBLISHED UNDER THE AUSPICES OF*

*THE BRISTOL MEDICO-CHIRURGICAL SOCIETY.*

EDITOR:

R. SHINGLETON SMITH, M.D., B.Sc.

WITH WHOM ARE ASSOCIATED

J. MICHELL CLARKE, M.A., M.D.

F. R. CROSS, M.B. AND W. H. HARSANT.

ASSISTANT-EDITOR:

L. M. GRIFFITHS.

"Scire est nescire, nisi id me

Scire alius sciret."

*VOL. XII.*

BRISTOL: J. W. ARROWSMITH;
LONDON: J. & A. CHURCHILL, 11 NEW BURLINGTON STREET.
1894.

L. H.

R 31
.B8

J.W. Arrowsmith

Publisher

Bristol

### CONTENTS OF VOLUME XII.

*Page*

Intussusception. By ARTHUR W. PRICHARD, M.R.C.S. Eng. ... ... 1

Appendicitis. By JAMES SWAIN, M.D., M.S. Lond., F.R.C.S. Eng. 9

So-called "Duct Cancer" of the Breast, with the Account of a Case of Large Recurrent Duct Papilloma. By CHARLES A. MORTON, F.R.C.S. Eng. ... ... ... ... ... ... ... ... ... ... ... ... 25

Somnambulism. By J. MICHELL CLARKE, M.A., M.D. Cantab., M.R.C.P. ... ... ... ... ... ... ... ... ... ... ... ... ... 81

Adherent Pericardium in Children. By THEODORE FISHER, M.D. Lond. ... ... ... ... ... ... ... ... ... ... ... ... ... ... 93

A Case of Syringomyelia. By F. H. EDGEWORTH, B.A., M.B. Cantab., B.Sc. Lond. ... ... ... ... ... ... ... ... ... ... 100

Pigmentation in Amenorrhœa. By A. E. AUST LAWRENCE, M.D. Aberd. ... ... ... ... ... ... ... ... ... ... ... ... ... 107

The Bristol Meeting of the British Medical Association, 1894 ... 161

Ergot of Rye as an Oxytocic. By J. G. SWAYNE, M.D. Lond. ... 225

Case of Myxœdema. By W. A. HAYES, M.R.C.S. Eng., L.R.C.P. Lond. ... ... ... ... ... ... ... ... ... ... ... ... ... ... 230

Two Cysts of Unusual Origin. By CHARLES A. MORTON, F.R.C.S. Eng. ... ... ... ... ... ... ... ... ... ... ... ... ... ... 232

Comparative Pathology. A Study in the Gardens of the Clifton Zoological Society. By A. J. HARRISON, M.B. Lond. ... ... 273

Cases of Senile Nerve Degeneration Resembling General Paralysis. By S. W. MACILWAINE, M.R.C.S. Eng., L.R.C.P. Lond. ... ... 294

Progress of the Medical Sciences ... ... ... 30, 108, 235, 297

Reviews of Books ... ... ... ... ... ... ... 45, 122, 250, 309

Notes on Preparations for the Sick ... ... ... 60, 143, 263, 326

Meetings of Societies ... ... ... ... ... ... ... 62, 149, 329

The Library of the Bristol Medico-Chirurgical Society ... ... ... ... ... ... ... ... ... 75, 152, 268, 330

The William F. Jenks Memorial Prize ... ... ... ... ... ... 154

The International Congress of Hygiene and Demography ... 154

Scraps ... ... ... ... ... ... ... ... ... ... 79, 155, 271, 335

### The Library of the Bristol Medico-Chirurgical Society.

*The following donations have been received since the publication of the list in December*:

*February* 28*th*, 1894.

| | | |
|---|---|---|
| The Dowager Lady Bowman (1) ... ... ... ... | 2 | volumes. |
| The Council of the Clinical Society (2) ... ... ... | 16 | ,, |
| L. M. Griffiths (3) ... ... ... ... ... ... .. | 3 | ,, |
| Christopher Martin, M.B. (4) ... ... ... ... ... | 1 | volume. |
| The Royal Academy of Medicine in Ireland (5) ... | 1 | ,, |
| R. Shingleton Smith, M.D. (6) ... ... ... ... ... ... | 1 | ,, |
| The Surgeon-General, United States Army (7) ... | 1 | ,, |
| The Editors of the Pathological Report of University College, London (8) ... ... ... ... ... | 1 | ,, |
| J. Wright & Co. (9) ... ... ... ... ... ... ... | 1 | ,, |

Unbound periodicals have been received from Dr. Shingleton Smith.

### ELEVENTH LIST OF BOOKS.

The figures in brackets refer to the figures after the names of the donors, and show by whom the volumes were presented. The books to which no such figures are attached have either been bought from the Library Fund or received through the *Journal.*

---

| | | | |
|---|---|---|---|
| **Allingham, H. W.** | *Internal Derangements of the Knee-Joint* | | 1889 |
| **Allingham, W.** | *Diseases of the Rectum.* 5th Ed. (Ed. by H. W. Allingham) | | 1888 |
| **Arlidge, J. T.** | *Diseases and Mortality of Occupations* | | 1892 |
| **Bastian, H. C.** | *The Brain as an Organ of Mind* | | 1880 |
| **Beasley, H.** | *The Druggist's General Receipt Book* | 9th Ed. | 1886 |
| ,, | *The Book of Prescriptions* | 7th Ed. | 1892 |
| **Bowlby, A. A.** | *Injuries and Diseases of Nerves* | | 1889 |
| **Bowman, Sir W.** | *Collected Papers.* (Ed. by J. Burdon-Sanderson and J. W. Hulke) | (1) 2 vols. | 1892 |
| **Boxall, R.** | *Antiseptics in Midwifery* | | 1894 |
| **Bramwell, B.** | *Atlas of Clinical Medicine.* Vol. II., Part III. | | 1893 |
| **Brewis, N. T.** | *Outlines of Gynæcological Diagnosis* | | 1894 |
| **Bristowe, J. S.** | *Diseases of the Nervous System* | | 1888 |
| **Brunton, T. L.** | *Disorders of Digestion* | | 1886 |
| **Buren, W. H. van** | *Diseases of the Rectum* | | 1881 |
| **Buzzard, T.** | *Paralysis from Peripheral Neuritis* | | 1886 |
| **Campbell, H.** | *Headache and other Morbid Cephalic Sensations* | | 1894 |
| **Charteris, M.** | *Health-Resorts at Home and Abroad* | 2nd Ed. | 1887 |
| **Cheadle, W. B.** | *Artificial Feeding of Infants* | 2nd Ed. | 1892 |
| **Clouston, T. S.** | *Clinical Lectures on Mental Diseases* | 3rd Ed. | 1892 |
| **Cooley, A. J.** | *A Cyclopædia of Practical Receipts.* 7th Ed. (Ed. by W. North.) 2 vols. | | 1892 |
| **Corfield, W. H.** | *Dwelling Houses* | 3rd Ed. | 1894 |
| **Coupland and O. Sturges, S.** | *Pneumonia* | 2nd Ed. | 1890 |
| **Dickinson, W. H.** | *The Tongue as an Indication in Disease* | | 1888 |
| **Donald, A.** | *An Introduction to Midwifery* | | 1894 |
| *Ear, Nose, and Throat, Diseases of the.* | (Ed. by C. H. Burnett.) | 2 vols. | 1893 |
| **Faulkner, A. S.** | *A Guide to the Public Medical Services* | | 1893 |
| **Fothergill, J. M.** | *Gout in its Protean Aspects* | | 1883 |
| ,, | *The Physiological Factor in Diagnosis* | 2nd Ed. | [1884] |
| **Goodhart, J. F.** | *On Common Neuroses* | 2nd Ed. | 1894 |
| **Hart, D. B.** | *Selected Papers in Gynæcology and Obstetrics* | | 1893 |
| **Hastings, J.** | *Pulmonary Consumption* | (3) | 1843 |
| **Haultain, F. W. N.** | *A Practical Handbook of Midwifery* | | 1894 |
| **Heyden, W. van der** | *Description of a newly-devised Sanitary Building* | | 1893 |

**Horne, J. F.** ... *Trephining in its Ancient and Modern Aspect* ... ... 1894
**Hwass, T.** ... ... *Studier öfver Transitorisk Albuminuri* ... ... ... ... 1893
*Hygiene and Diseases of Warm Climates.* (Ed. by A. Davidson) ... ... 1893
**Inglis-Parsons, J.** *The Healing of Rodent Cancer by Electricity* ... ... ... 1893
**Jacob, A.** ... ... *On Cataract* ... ... ... ... ... ... ... (3) 1851
**Jacobson, W. H. A.** *Diseases of the Male Organs of Generation* ... ... ... 1893
**Jeaffreson, C. S.**... *Nursing in Eye Diseases* ... ... ... ... ... ... ... 1894
**Jessett, F. B.** ... *Cancer of the Mouth, Tongue, and Œsophagus* ... ... 1892
,, ... ... *Surgical Diseases of the Stomach* ... ... ... ... ... 1892
**Jones, T.** ... ... *Diseases of the Bones* ... ... ... ... ... ... ... 1887
*Library of the Surgeon-General's Office, United States Army, Index-Catalogue of* ... ... ... ... .. (7) Vol. XIV. 1893
**McCaw, J.** ... ... *Aids to the Treatment of Diseases of Children (Medical)* [1893]
**MacCormac, Sir W.** *Surgical Operations.* Part II., *Amputations, &c.* ... 1889
**Macewen, W.** ... *Pyogenic Infective Diseases of the Brain and Spinal Cord* 1893
**Malcolm, J. D.** ... *The Physiology of Death from Traumatic Fever* ... ... 1893
**Manley, T. H.** ... *Hernia* ... ... ... ... ... ... ... ... ... 1893
**Marsh, H.** ... ... *Diseases of the Joints* ... ... ... ... ... ... ... 1886
**Martin, B. R.** ... *Diphtheria and its Treatment* ... ... ... ... ... ... 1894
**Martin, C.** ... ... *The After-Treatment of Cases of Abdominal Section* ... 1894
,, ... ... *The Nerve Theory of Menstruation*... ... ... ... (4) 1893
**Mickle, W. J.** ... *General Paralysis of the Insane* ... ... ... 2nd Ed. 1886
**Morris, M.** ... ... *Diseases of the Skin*... ... ... ... ... ... ... ... 1894
**Moullin, C. W. M.** *Sprains* ... ... ... ... ... ... ... 2nd Ed. 1894
**Muskett, P. E.** ... *The Art of Living in Australia* ... ... ... ... .. [1893]
**Newman, D.** ... *Diseases of the Kidney* ... ... ... ... ... ... ... 1888
**Owen, E.** ... ... *The Surgical Diseases of Children* .. ... .. 2nd Ed. 1889
**Paget, Sir J.** ... *Studies of Old Case-Books* ... ... ... ... ... ... 1891
**Parker and H. O. Thomas, R.** *Intestinal Obstruction and Abdominal Hernia* 1883
**Parker, R. W.** ... *Diphtheria* ... ... ... ... ... ... ... 3rd Ed. 1891
**Parkes, L. C.** ... *Infectious Diseases* ... ... ... ... ... ... ... ... 1894
**Pekelharing and C. Winkler, C. A.** *Beri-Beri.* (Tr. by J. Cantlie) ... 1893
*Pharmacopœia of the United States of America, The* ... ... ... ... ... 1893
**Phillips, L.** ... *Medicated Baths in Skin Diseases* ... ... ... ... ... 1893
**Roberts, Sir W.**... *Digestion and Diet* ... ... ... ... ... ... ... ... 1891
**Roose, R.** ... ... *Gout and its Relations to Diseases of the Liver and Kidneys* ... ... ... ... ... ... ... (3) 4th Ed. 1887
**Roth, B.** ... ... *Lateral Curvature of the Spine* ... ... ... ... ... 1889
**Skene, A. J. C.** ... *Diseases of Women* ... ... ... ... ... ... 2nd Ed. 1892
**Smith, E.** ... ... *Disease in Children* ... ... ... ... ... ... 2nd Ed. 1889
**Stewart, W. R. H.** *Aids to Otology* ... ... ... ... ... ... ... 2nd Ed. [1893]
**Sturges, O.** ... *Chorea* ... ... ... ... ... ... ... ... ... 2nd Ed. 1893
,, **and S. Coupland** *Pneumonia* ... ... ... ... ... ... ... 2nd Ed. 1890
**Talamon, C.** ... *Appendicitis and Perityphlitis.* (Tr. by R. J. A. Berry) 1893
**Thomas and R. Parker, H. O.** *Intestinal Obstruction and Abdominal Hernia* 1883
**Thomas, T. G.** ... *The Diseases of Women.* 6th Ed. (Ed. by P. F. Mundé) ... ... ... ... ... ... ... ... ... ... 1891
**Thorne, W. B.** ... *The Open-Air Treatment of Phthisis* ... ... ... ... 1849
**Winkler and C. A. Pekelharing, C.** *Beri-Beri.* (Tr. by J. Cantlie) ... 1893

### TRANSACTIONS, REPORTS, JOURNALS, &c.

| | | |
|---|---|---|
| American Journal of Obstetrics, The | Vol. XXVIII. | 1893 |
| American Journal of the Medical Sciences, The | Vol. CVI. | 1893 |
| American Practitioner and News, The | Vols. XV., XVI. | 1893 |
| American Surgical Association, Transactions of the | Vol. XI. | 1893 |
| Archives cliniques de Bordeaux | Tome II. | 1893 |
| Birmingham Medical Review, The | Vol. XXXIV. | 1893 |
| Bristol and Clifton Directory | (9) | 1894 |
| British Medical Journal | Vol. II. for | 1893 |
| Clinical Society's Transactions | (2) Vols. XI.—XXVI. | 1878–93 |
| Deutsche medicinische Wochenschrift | | 1893 |
| Dublin Journal of Medical Science, The | Vol. XCVI. | 1893 |
| Epitome of Medicine, The | Vol. X. | 1893 |
| Fortschritte der Krankenpflege | | 1893 |
| Gazette des Hôpitaux | | 1893 |
| Glasgow Medical Journal, The | Vol. XL. | 1893 |
| Hazell's Annual | | 1894 |
| Hospital, The | Vol. XIV. | 1893 |
| Hospitals— | | |
| Johns Hopkins Hospital Reports. | Vol. III., Nos. 7, 8, 9 | 1894 |
| University College, London. Pathological Report | (8) Vol. II. | 1893 |
| International Medical Magazine | Vol. I. | 1893 |
| Journal of Cutaneous and Genito-Urinary Diseases | Vol. XI. | [1893] |
| Journal of Laryngology, Rhinology, and Otology, The | Vol. VII. | 1893 |
| Journal of Physiology, The | (6) Vol. XIII. | 1892 |
| Lancet, The | Vol. II. for | 1893 |
| Liverpool Medico-Chirurgical Journal, The | Vol. XIII. | 1893 |
| Medical Age, The | | 1893 |
| Medical and Surgical Reporter, The | Vol. LXVIII. | 1893 |
| Medical Directory, The | | 1894 |
| Medical News, The | Vol. LXIII. | 1893 |
| Medical Press and Circular, The | Vol. CVII. | 1893 |
| Medical Record | Vol. XLIV. | 1893 |
| New York Academy of Medicine, Transactions of the | Vol. IX. | 1893 |
| New York Medical Journal, The | Vol. LVIII. | 1893 |
| Retrospect of Medicine, Braithwaite's | Vol. CVIII. | 1894 |
| Revue de Laryngologie, d'Otologie et de Rhinologie | Tome XIII. | 1893 |
| Royal Academy of Medicine in Ireland, Transactions of the | (5) Vol. XI. | 1893 |
| Sheffield Medical Journal, The | Vol. I. | [1893] |
| Therapeutic Gazette, The | Vol. XVII. | 1893 |
| Union médicale, L' | Tome LVI. | 1893 |
| West London Medico-Chirurgical Society, Proceedings of the | Vol. V. | 1893 |
| Whitaker's Almanack | | 1894 |
| Year-Book of Treatment, The | | 1894 |

### Bristol Medico-Chirurgical Society.

### President.

J. GREIG SMITH, M.B., C.M., M.A. Aberd., F.R.S.E.

### President-Elect.

A. J. HARRISON, M.B. Lond.

### Honorary Secretary.

G. MUNRO SMITH.

### Committee.

*Past Presidents.*

R. W. COE.
AUGUSTIN PRICHARD, M.D. Berlin.
J. G. SWAYNE, M.D. Lond.
E. LONG FOX, M.D. Oxon.
JAMES G. DAVEY, M.D. St. And.
W. JOHNSTONE FYFFE, M.D., B.A. Dub.
W. H. SPENCER, M.D., M.A. Cantab.
F. POOLE LANSDOWN.
L. M. GRIFFITHS.
R. SHINGLETON SMITH, M.D., B.Sc. Lond.
NELSON C. DOBSON.
S. H. SWAYNE.
F. R. CROSS, M.B. Lond.
E. MARKHAM SKERRITT, M.D., B.S., B.A. Lond.

J. MICHELL CLARKE, M.D., M.A. Cantab.
JOHN DACRE.
W. H. HARSANT.
A. E. AUST LAWRENCE, M.D., C.M. Aberd.
ARTHUR W. PRICHARD.
J. E. SHAW, M.B., C.M. Ed.

Medical Men wishing to join the Society are requested to communicate with the Honorary Secretary, G. Munro Smith, 28 Alma Road, Clifton, Bristol. The Annual Subscription is 21/-.

---

*Extracts from Journal By-laws.*

4.—The *Journal* shall be delivered free to Subscribers and also to Members of the Society whose subscriptions are not in arrear.

6.—The Annual Subscription to the *Journal* for those who pay before the 1st of July shall be 5/-, post free, or 6/- if paid after that date.

---

Communications intended for insertion in the *Journal* should be sent to the Editor, Dr. Shingleton Smith, Deepholm, Clifton Park, Clifton, Bristol.

Books for Review, Exchange Journals, and communications referring to the delivery of the *Journal* should be sent to the Assistant-Editor, L. M. Griffiths, 9 Gordon Road, Clifton, Bristol, to whom Subscriptions are to be paid *in advance*.

Cases for Binding Vols. I.–XI. are now ready, and may be obtained, price 7*d*. each (postage 2*d*. extra), upon application to J. W. Arrowsmith, Quay Street, Bristol; or the *Journal* will be bound, including Case, for 1/3 per volume.

### Bristol Medico-Chirurgical Society.

---

### PAST PRESIDENTS.

1874—75. FREDERICK BRITTAN, M.D., B.A. Dub.

1875—76. ROBERT WILLIAM COE.

1876—77. SAMUEL MARTYN, M.D. Ed.

1877—78. AUGUSTIN PRICHARD, M.D. Berlin.

1878—79. HENRY EDWARD FRIPP, M.D. Würzburg.

1879—80. WILLIAM MICHELL CLARKE.

1880—81. JOSEPH GRIFFITHS SWAYNE, M.D. Lond.

1881—82. EDWARD LONG FOX, M.D. Oxon.

1882—83. JAMES GEORGE DAVEY, M.D. St. And.

1883—84. WILLIAM JOHNSTONE FYFFE, M.D., B.A. Dub.

1884—85. GEORGE FORSTER BURDER, M.D. Aberd.

1885—86. WILLIAM HENRY SPENCER, M.D., M.A. Cantab.

1886—87. FRANCIS POOLE LANSDOWN.

1887—88. LEMUEL MATTHEWS GRIFFITHS.

1888—89. ROBERT SHINGLETON SMITH, M.D., B.Sc. Lond.

1889—90. NELSON CONGREVE DOBSON.

1890—91. SAMUEL HENRY SWAYNE.

1891—92. FRANCIS RICHARDSON CROSS, M.B. Lond.

1892—93. EDWARD MARKHAM SKERRITT, M.D., B.S., B.A. Lond.

1894.

### LIST OF SUBSCRIBERS

TO THE

### Bristol Medico-Chirurgical Journal.

*Those marked * are Members of the Society.*

| | |
|---|---|
| Ackland, J. McKno ... ... ... ... ... ... | 24 Southernhay, Exeter. |
| *Ackland, W. R. ... ... ... ... ... ... ... ... | 5 Rodney Place, Clifton. |
| Adams, George ... ... ... ... ... ... ... | 11 Westbourne Place, Clifton. |
| *Adams, John Barfield ... ... ... ... ... ... | 110 Cotham Road, Bristol. |
| Alexander, James, M.D., M.Ch., B.A. Dub. | Bishop's Place, Paignton. |
| Aldridge, Charles, M.D., C.M. Aberd. ... | Plympton. |
| Aldous, George F. ... ... ... ... ... ... | Charlton House, Mannamead, Plymouth. |
| *Alford, George Ernest ... ... ... ... ... | 5 Royal Crescent, Weston-super-Mare. |
| Alford, Henry J., M.D. Lond. ... ... ... | 1 Hovelands Terrace, Taunton. |
| Anstie, Thomas B. ... ... ... ... ... ... ... | 21 Northgate Street, Devizes. |
| *Atchley, George F., M.B. Lond.... ... ... | Tyndall's Park, Bristol. |
| Avarne, A. B. ... ... ... ... ... ... ... ... ... | Blaenavon, Monmouthshire. |
| *Aveline, H. T. S.... ... ... ... ... ... ... | Asylum, Stapleton, Gloucestershire. |
| | |
| Baker, A. de W. ... ... ... ... ... ... ... ... | 2 Lawn Terrace, Dawlish. |
| Ballantyne, J. W., M.D., C.M. Ed.... ... ... | 24 Melville Street, Edinburgh. |
| Bannatyne, Gilbert A., M.D., C.M. Glasg.... | 1 Paragon, Bath. |
| *Barclay, W. M. ... ... ... ... ... ... ... ... | Queen's Road, Clifton. |
| Barker, Arthur E. ... ... ... ... ... ... ... | 87 Harley Street, London, W. |
| Baretti, T. G. L'Enardi ... ... ... ... ... | 49 Royal York Crescent, Clifton. |
| *Baron, Barclay J., M.B., C.M. Ed. ... ... | 16 White Ladies Road, Clifton. |
| Barr, J. Coleman ... ... ... ... ... ... ... ... | 2 Cranmore Cottages, Aldershot. |
| *Basset, Walter ... ... ... ... ... ... ... ... | 24 Stow Hill, Newport, Mon. |
| *Bateman, F. J. B. ... ... ... ... ... ... ... | Alburgh House, Wells, Somersetshire. |
| Batten, Rayner W., M.D. Lond. ... ... ... | 1 Brunswick Square, Gloucester. |
| Batten, W. Smith ... ... ... ... ... ... ... | Curzon House, Calne. |
| *Beddoe, John, M.D. Ed., B.A. Lond., F.R.S. | The Chantry, Bradford-on-Avon. |
| Benham, Harry A., M.D., C.M. Aberd. ... | Asylum, Stapleton, Gloucestershire. |
| Bennett, W. S. ... ... ... ... ... ... ... ... | Escot, Penzance. |
| *Benson, A. H. ... ... ... ... ... ... ... ... | Wrington, Somersetshire. |
| Berry, James, M.B., B.S. Lond.... ... ... ... | 60 Welbeck Street, London, W. |
| Bevan, W. L. P., M.D., C.M. Ed. ... ... ... | Alton, Hampshire. |
| *Blacker, A. E. ... ... ... ... ... ... ... ... | Richmond Hill Avenue, Clifton. |
| *Blacker, E. ... ... ... ... ... ... ... ... ... | 12 White Ladies Road, Clifton. |
| *Blagg, Arthur F. ... ... ... ... ... ... ... | 6 West Mall, Clifton. |
| Blomfield, Arthur G., M.D. Aberd. ... ... | 34 West Southernhay, Exeter. |
| *Board, Edmund C. ... ... ... ... ... ... ... | 11 Caledonia Place, Clifton. |
| *Boyce, James G. ... ... ... ... ... ... ... ... | 6 Colston Parade, Bristol. |
| Boys, Arthur Henry ... ... ... ... ... ... | Chequer Street, St. Alban's. |

### LIST OF SUBSCRIBERS.

| | |
|---|---|
| Brabazon, A. B., M.D. Aberd. ... ... ... ... | 12 Darlington Street, Bath. |
| *Brasher, Charles W. J. ... ... ... ... | 144 Cheltenham Road, Bristol. |
| *Bright, J. A. ... ... ... ... ... ... ... ... ... ... | Glastonbury. |
| *Broom, John, M.D., Ch.D., Obst.D. Brussels | St. Paul's Road, Clifton. |
| Brown, G. Arthur ... ... ... ... ... ... ... | Tredegar. |
| Brown, William, M.D., C.M. Glasg. ... ... | Fishponds, Gloucestershire. |
| Browne, A. E. Newbury ... ... ... ... ... ... | Burbage, Wiltshire. |
| Browne, G. Henry ... ... ... ... ... ... ... | The Hermitage, Brynmawr, Breconshire. |
| Browne, J. Walton, M.D., B.A. Roy. Univ. I. | 10 College Square North, Belfast. |
| Burges, R. E., M.D., M.Ch., B.A. Roy. Univ. I. | 3 Egerton Terrace, Hoole Road, Chester. |
| Burroughs, E. F. H. ... ... ... ... ... ... ... | 3 Elgin Park, Bristol. |
| *Bush, J. Paul ... ... ... ... ... ... ... ... ... | Clifton Park, Clifton. |
| | |
| *Cameron, John, M.B., C.M. Glasg. ... ... | Ashley Hill, Bristol. |
| Campbell, George... ... ... ... ... ... ... ... | Chilton Polden. |
| Carolin, George ... ... ... ... ... ... ... ... | Stonehouse, Gloucestershire. |
| *Carr, Alexander ... ... ... ... ... ... ... ... | 150 Wells Road, Bristol. |
| *Carter, T. M. ... ... ... ... ... ... ... ... ... | 13 Dowry Square, Clifton. |
| *Carter, W. F.... ... ... ... ... ... ... ... ... | 48 Coronation Road, Bristol. |
| Cave, Edward J., M.D. Lond. ... ... ... ... | Crewkerne. |
| Cheves, Alexander B., M.D., M.A. Aberd. ... | Millbrook, Devonshire. |
| *Clarke, John Michell, M.D., M.A. Cantab. | 28 Pembroke Road, Clifton. |
| Clay, Challoner ... ... ... ... ... ... ... ... | Manor House, Fovant. |
| Clay, R. Hogarth, M.D. Ed. ... ... ... ... | 4 Windsor Villas, Plymouth. |
| Close, John B., M.B. Dur. ... ... ... ... | 61 Spring Bank, Hull. |
| Coates, Harcourt ... ... ... ... ... ... ... | 17 Endless Street, Salisbury. |
| Coathupe, Edwin W. ... ... ... ... ... ... ... | 7 Clifton Park, Clifton. |
| Cobbold, C. S. W., M.D. Würzburg ... ... | Bailbrook House, Bath. |
| *Coe, R. W. ... ... ... ... ... ... ... ... ... | 7 Pembroke Road, Clifton. |
| Coker, T. V. ... ... ... ... ... ... ... ... ... ... | 2 Chesterfield Place, Clifton. |
| Cole, Thomas, M.D. Lond. ... ... ... ... | 18 Circus, Bath. |
| *Collins, H. W. ... ... ... ... ... ... ... ... | Wrington, Somersetshire. |
| Colman, Henry ... ... ... ... ... ... ... ... | Pewsey, Wiltshire. |
| Colman, Thomas J., M.D. Ed.... ... ... ... | Elmgrove Road, Bristol. |
| Colmer, Ptolemy S. H., M.D. Dur. ... ... | South Street House, Yeovil. |
| *Cook, Henri, M.B., C.M. Aberd. ... ... ... | 45 Stackpool Road, Bristol. |
| Cooke, A. S. ... ... ... ... ... ... ... ... ... ... | Badbrook House, Stroud. |
| *Corbould, George G. ... ... ... ... ... ... | 22 Berkeley Square, Bristol. |
| Cossham, W. R., M.D., C.M. Aberd.... ... ... | Cirencester. |
| *Cotton, William, M.B., C.M., M.A. Ed. ... | 227 Gloucester Road, Bristol. |
| Cousins, J. Ward, M.D. Lond. ... ... ... | Kent Road, Southsea. |
| Cowan, F. S. ... ... ... ... ... ... ... ... ... | 27 Queen Square, Bath. |
| Cox, E. Owen ... ... ... ... ... ... ... ... ... | Gillingham, Dorsetshire. |
| Craddock, Samuel ... ... ... ... ... ... ... | South Lyncombe, Bath. |
| Crespi, Alfred J. H. ... ... ... ... ... ... | Poole Road, Wimborne. |
| *Cross, F. Richardson, M.B. Lond. ... ... | Worcester House, Clifton. |
| *Crossman, Edward, M.D. Dur. ... ... ... | Hambrook, Gloucestershire. |
| *Crouch, C. Percival, M.B. Lond. ... ... ... | 3 Princes Buildings, Weston-super-Mare. |
| Culross, James, M.B., C.M., M.A. Glasg. ... | 66 Queen Street, Newton Abbot. |
| Cunningham, C. L. ... ... ... ... ... ... ... | Chudleigh. |
| | |
| *Dacre, John ... ... ... ... ... ... ... ... ... | 14 Eaton Crescent, Clifton. |
| Daniel, T. P. ... ... ... ... ... ... ... ... ... | Beaminster, Dorsetshire. |
| *Davey, James G., M.D. St. And. ... ... ... | 31 Avonmore Road, London, W. |
| *Davies, D. S., M.D. Lond. ... ... ... ... | 60 Oakfield Road, Clifton. |
| Davies, Ebenezer... ... ... ... ... ... ... ... | Brunswick House, Swansea. |
| Davies, H. N. ... ... ... ... ... ... ... ... ... | Porth, Glamorganshire. |
| Davis, Robert ... ... ... ... ... ... ... ... | Oakleigh, Epsom. |
| Davis, Theodore, M.D. Lond. ... ... ... | Beachcroft, Clevedon. |

### LIST OF SUBSCRIBERS.

| | |
|---|---|
| Davy, Henry, M.D. Lond. ... ... ... ... ... | Southernhay House, Exeter. |
| Day, Percy Howard ... ... ... ... ... ... ... | Stalmine, Poulton-le-Fylde. |
| Deas, P. Maury, M.B., M.S. Lond. ... ... | Wonford House, Exeter. |
| Dening, Edwin ... ... ... ... ... ... ... ... | Manor House, Stow-on-the-Wold. |
| *Devis, Harry F. ... ... ... ... ... ... ... | Wells Road, Bristol. |
| Dickie, James Steel, M.D., C.M. Aberd. ... | Christchurch Road, Bournemouth. |
| *Dobson, Nelson C. ... ... ... ... ... ... | 27 Victoria Square, Clifton. |
| Domville, Edward J. ... ... ... ... ... ... ... | 44 Southernhay, Exeter. |
| *Dowson, W., M.D., B.C., M.A. Cantab. ... ... | Great George Street, Bristol. |
| Doyne, Robert W. ... ... ... ... ... ... ... | 70 Woodstock Road, Oxford. |
| Duffett, E. D. ... ... ... ... ... ... ... ... | 5 London Street, Swindon, Wiltshire. |
| Duke, Alexander ... ... ... ... ... ... ... ... | Alconbury, Cheltenham. |
| Dunbar, Eliza L. Walker, M.D. Zürich ... | 9 Oakfield Road, Clifton. |
| *Duncan, William ... ... ... ... ... ... ... | Westbury-on-Trym. |
| Eadon, W. F. Bailey ... ... ... ... ... ... ... | Hambrook, Gloucestershire. |
| *Eager, Reginald, M.D. Lond. ... ... ... | Northwoods, Winterbourne. |
| Eddowes, Charles ... ... ... ... ... ... ... | Maddington, Devizes. |
| *Edgeworth, F. H., M.B., B.C., B.A. Cantab., B.Sc. Lond. ... ... ... ... ... ... ... ... ... | 1 Richmond Hill, Clifton. |
| Edis, Thomas ... ... ... ... ... ... ... ... ... | 66 Barton Street, Gloucester. |
| Edwards, W. T., M.D. Lond. ... ... ... ... | 129 Queen Street, Cardiff. |
| Elder, William ... ... ... ... ... ... ... ... | 7 Trelawney Road, Bristol. |
| *Elliott, Christopher, M.D., B.A. Dub. ... | 88 Pembroke Road, Clifton. |
| Ellis, W. McD., M.D. Brussels ... ... ... | 7 Alexander Buildings, Bath. |
| Elsworth, R. C., M.B., C.M. Ed. ... ... ... | 203 St. Helen's Road, Swansea. |
| Embleton, Dennis C. ... ... ... ... ... ... | St. Michael's Road, Bournemouth. |
| *Etches, W. R. ... ... ... ... ... ... ... | Macclesfield. |
| Evans, J. Fenton, M.B., C.M. Ed. ... ... | 6 Whitehall Yard, London, S.W. |
| Evans, W. G. ... ... ... ... ... ... ... ... ... | Beckington. |
| *Ewens, John ... ... ... ... ... ... ... ... | 61 Cotham Hill, Bristol. |
| Fairbanks, W., M.D., C.M. Ed. ... ... ... | Wells, Somersetshire. |
| *Fendick, R. George ... ... ... ... ... ... | St. Paul's Road, Clifton. |
| *Fenton, Henry ... ... ... ... ... ... ... | 19 Caledonia Place, Clifton. |
| Finch, Thomas, M.B., B.A. Cantab.... ... ... | Watcombe View, Babbacombe. |
| Finch, Thomas, M.D. St. And. ... ... ... | Westville, St. Mary Church. |
| *Firth, J. L., M.D., B.S. Lond.... ... ... ... | General Hospital, Bristol. |
| Fisher, Fred. B. ... ... ... ... ... ... ... ... | West Walk, Dorchester. |
| *Fisher, Theodore, M.D. Lond. ... ... ... | 25 Pembroke Road, Clifton. |
| *Flemming, C. E. S. ... ... ... ... ... ... | Freshford. |
| Ford, Joseph ... ... ... ... ... ... ... ... ... | Wedmore. |
| Forty, D. H. ... ... ... ... ... ... ... ... ... | Wotton-under-Edge. |
| Fox, Arthur E. W., M.B., C.M. Ed. ... ... | 16 Gay Street, Bath. |
| *Fox, Bonville B., M.D., M.A. Oxon. ... ... | Brislington. |
| Fox, Charles Henry, M.D. St. And. ... ... | Brislington. |
| Fox, E. Lawrence, M.D., B.Ch , M.A. Cantab. | 19 Lockyer Street, Plymouth. |
| *Fox, E. Long, M.D. Oxon. ... ... ... ... | Church House, Clifton. |
| Fox, John Alfred... ... ... ... ... ... ... ... | Morrab Road, Penzance. |
| Fraser, Græme B. ... ... ... ... ... ... ... | Beach Road, Weston-super-Mare. |
| Freeman, Henry W. ... ... ... ... ... ... | 24 Circus, Bath. |
| Fry, J. Blount ... ... ... ... ... ... ... ... | Ashley Lodge, Esher. |
| *Fyffe, W. Johnstone, M.D., B.A. Dub. ... | 2 Rodney Place, Clifton. |
| Gamble, C. H.... ... ... ... ... ... ... ... ... | Barnstaple. |
| George, R. Julyan, M.B., C.M. Ed. ... ... | Port Isaac, Cornwall. |
| *Gibbs, Alfred N. Godby ... ... ... ... ... | 52 Whiteladies Road, Clifton. |
| Gill, John, M.D. Brussels ... ... ... ... ... | 75 Pembroke Road, Clifton. |

### LIST OF SUBSCRIBERS.

| | |
|---|---|
| Gloag, George A. ... ... ... ... ... ... | 7 College Green, Bristol. |
| Goodfellow, W. R. ... ... ... ... ... ... ... | Roche, Cornwall. |
| Goodridge, Henry F. A., M.D. Lond.... ... | 10 Brock Street, Bath. |
| Goodwyn, A. H. ... ... ... ... ... ... ... ... | Droxford, Hampshire. |
| Gordon, W., M.B., B.C., M.A. Cantab.... ... | Barnfield Lodge, Exeter. |
| Goss, T. Biddulph ... ... ... ... ... ... ... | 1 Circus, Bath. |
| Grace, Alfred ... ... ... ... ... ... ... ... | Chipping Sodbury. |
| Grace, Edward Mills ... ... ... ... ... ... | Thornbury, Gloucestershire. |
| Grace, Henry ... ... ... ... ... ... ... ... | Kingswood, Bristol. |
| Grace, W. G. ... ... ... ... ... ... ... ... ... | 15 Victoria Square, Clifton. |
| Granville, J. Mortimer, M.D. St. And. ... | 14 Hanover Square, London, W. |
| Gratte, C. Brooke ... ... ... ... ... ... ... | 3 Lansdowne Place, Newport, Mon. |
| Gray, A. Murray ... ... ... ... ... ... ... ... | 17 Market Place, Devizes. |
| Grey, T. C. ... ... ... ... ... ... ... ... ... | The Shrubbery, Weston-super-Mare. |
| *Griffiths, C. A. ... ... ... ... ... ... ... | General Hospital, Swansea. |
| *Griffiths, J. S. ... ... ... ... ... ... ... | 25 Redland Park, Bristol. |
| *Griffiths, L. M. ... ... ... ... ... ... ... ... | 9 Gordon Road, Clifton. |
| Grote, G. W., M.B. Toronto ... ... ... ... | 54 Terrace Road, London, E. |
| Groves, J., M.B., B.A. Lond. ... ... ... ... | Carisbrooke. |
| *Hailes, Clements, D. G., M.D., C.M. Ed.... | 9 King's Parade, Clifton. |
| *Haines, A. H. ... ... ... ... ... ... ... ... ... | 8 Cotham Road, Bristol. |
| Hale, Edmund T. ... ... ... ... ... ... ... ... | The Grange, Chew Magna. |
| Halpin, W. C. ... ... ... ... ... ... ... ... ... | Parkend. |
| Hamlen-Williams, T. R. ... ... ... ... ... ... | Verlands, Cowbridge. |
| Handfield-Jones, Montagu, M.D. Lond. ... | 35 Cavendish Square, London, W. |
| Hardwick, A., M.B. Dur. ... ... ... ... ... ... | Newquay, Cornwall. |
| Harper, Joseph ... ... ... ... ... ... ... ... | Bear Street, Barnstaple. |
| *Harrison, A. J., M.B. Lond. ... ... ... ... | Guthrie Road, Clifton. |
| Harrison, Charles ... ... ... ... ... ... ... | Keynsham, Somersetshire. |
| Harsant, J. G., M.D., B.S. Lond. ... ... ... | Exeter Road, Bournemouth. |
| *Harsant, W. H. ... ... ... ... ... ... ... ... | 16 Pembroke Road, Clifton. |
| *Hawkins-Ambler, G. A. ... ... ... ... ... ... | 162 Upper Parliament Street, Liverpool. |
| Hawkins, Cæsar F. ... ... ... ... ... ... ... | North Petherton. |
| Haydon, Edgar, M.B., C.M. Glasg. ... ... | The Laurels, Newton Abbot. |
| Hayes, H. W. McCaulty ... ... ... ... ... | 5 Ilchester Gardens, London, W. |
| Hayes, W. A. ... ... ... ... ... ... ... ... ... | Elm Grove, Calne. |
| Hayman, Alfred G. ... ... ... ... ... ... ... | 1 Pembroke Road, Clifton. |
| *Hayman, C. A. ... ... ... ... ... ... ... ... ... | 17 Victoria Square, Clifton. |
| Heane, W. C. ... ... ... ... ... ... ... ... ... | Cinderford. |
| *Heaven, John C. ... ... ... ... ... ... ... ... | 2 Queen Square, Bristol. |
| *Herapath, C. K. C. ... ... ... ... ... ... ... | Cotham Park, Bristol. |
| *Hetling, H. E. ... ... ... ... ... ... ... ... | Kingsley Road, Bristol. |
| Hill, F. A., M.D. Brussels... ... ... ... ... | Thorncombe. |
| Hill, Hedley ... ... ... ... ... ... ... ... ... | 1 Redcliff Hill, Bristol. |
| Hills, Frederic F. ... ... ... ... ... ... ... | 146 Stapleton Road, Bristol. |
| Hoblyn, Francis Parker ... ... ... ... ... | 10 Bladud Buildings, Bath. |
| *Hoffman, A. W. W. ... ... ... ... ... ... ... | 5 Rodney Place, Clifton. |
| Hopkins, H. Culliford ... ... ... ... ... ... | 6 Bladud Buildings, Bath. |
| Horsfall, John, M.A. Oxon. ... ... ... ... | Richmond Hill, Bournemouth. |
| Hospital, Bristol General (*Sec.*, W. Thwaites). | |
| Hospital, Cincinnati, U.S.A. | |
| Hospital, Taunton and Somerset (*House-Surgeon*, J. G. Bain). | |
| Howells, William, M.B., C.M. Glasg. ... | Church House, Talgarth, Breconshire. |
| Howse, William ... ... ... ... ... ... ... ... | 8 London Street, Swindon, Wiltshire. |
| Howsin, E. Arthur, M.D. St. And. ... ... | Stroud. |
| Hudson, F. Horace ... ... ... ... ... ... ... | Sturminster Newton. |
| Hudson, H. R. ... ... ... ... ... ... ... ... | 182 Commercial Road, Newport, Mon. |
| Huggard, W. R., M.D., M.Ch., M.A. Roy. Univ. I. | Davos-Platz, Switzerland. |

### LIST OF SUBSCRIBERS.

| | |
|---|---|
| HUNT A. D. ... ... ... ... ... ... ... ... | Millbrook House, Chagford. |
| HYATT, J. T. ... ... ... ... ... ... ... ... | Shepton Mallet. |
| | |
| *IMLAY, G. A., M.B., C.M. Aberd. ... ... ... | 1 Cotham Park, Bristol. |
| INSTITUTION, LIVERPOOL MEDICAL (*Resident Librarian*, WILLIAM JONES). | |
| IRWIN, ALAN M. ... ... ... ... ... ... ... | Blakeney, Gloucestershire. |
| IVES, WILLIAM R. Y. ... ... ... ... ... ... | Portswood Road, Southampton. |
| | |
| JACKSON, J. B., M.D., M.Ch.Roy.Univ.I. ... | New York, U.S.A. |
| JACKSON, MARK, M.D. Roy. Univ. I.... ... ... | Bear Street, Barnstaple. |
| JACOBSON, W. H. A., M.B., M.C., M.A. Oxon. | 66 Great Cumberland Place, London, W. |
| JAMES, J. BOWEN ... ... ... ... ... ... ... | Woodlands, Aberaman, Aberdare. |
| *JAMISON, A., M.B., B.Ch., M.A. Roy. Univ. I. | Children's Hospital, Bristol. |
| JESSOP, CHARLES MOORE ... ... ... ... ... | Clare Lodge, Redhill. |
| *JOHNSTON, D. G., M.B., C.M. Glasg. ... ... | 92 Hampton Road, Bristol. |
| JONES, A. ORLANDO, M.D., C.M. Aberd. ... | Harrogate. |
| JONES, E. R. ... ... ... ... ... ... ... ... ... | Cilgynlle-fawr, New Quay, Cardiganshire. |
| JONES, EVAN ... ... ... ... ... ... ... ... ... | Ty-mawr, Aberdare. |
| JONES, H. MACNAUGHTON, M.D., M.Ch. Roy. Univ. I. ... ... ... ... ... ... ... ... ... ... | 141 Harley Street, London, W. |
| JONES, W. R., M.D., C.M. Glasg. ... ... ... | Senny Bridge, Breconshire. |
| JONES, W. W., M.B., C.M. Ed. ... ... ... | 47 Wellington Street, Merthyr Tydvil. |
| | |
| *KELLY, T. B. ... ... ... ... ... ... ... ... ... | Eye Hospital, Bristol. |
| *KEMM, F. ST. J.... ... ... ... ... ... ... ... | Worle. |
| KEMPE, ARTHUR W., M.D. Brussels ... ... | 14 Southernhay, Exeter. |
| KENDALL, BERNARD C.... ... ... ... ... ... | Elm Grove, Penally, Pembrokeshire. |
| KENNEDY, W. P., M.D., B.Ch., B.A. Dub. ... | High Street, Lydney. |
| KENT-JONES, D. ... ... ... ... ... ... ... | Plascoed, Deri. |
| KING, LOUIS J.... ... ... ... ... ... ... ... ... | 10 Russel Street, Bath. |
| KINNEIR, R. ... ... ... ... ... ... ... ... ... | The Tower House, Malmesbury. |
| KITSON, F. P. ... ... ... ... ... ... ... ... ... | Beaminster, Dorsetshire. |
| | |
| *LACE, F. ... ... ... ... ... ... ... ... ... ... | 5 Paragon, Bath. |
| LAING, W. A. GORDON, M.D., C.M. Glasg. ... | Mevagissey, St. Austell. |
| *LANE, HUGH ... ... ... ... ... ... ... ... | 11 Circus, Bath. |
| *LANSDOWN, F. POOLE ... ... ... ... ... ... | 19 White Ladies Road, Clifton. |
| *LANSDOWN, R. G. P., M.B., B.S. Dur. ... ... | 39 Oakfield Road, Clifton. |
| LAUWERS, DR. .. ... ... ... ... ... ... ... | Courtrai, Belgium. |
| *LAWRENCE, A. E. AUST, M.D., C.M. Aberd. . | 19 Richmond Hill, Clifton. |
| *LAWRENCE, HENRY ... ... ... ... ... ... | 19 Park Row, Bristol. |
| LAWRIE, J. MACPHERSON, M.D., C.M. Glasg. | Greenhill, Weymouth. |
| LAXTON, T. L.... ... ... ... ... ... ... ... | 4 Park Place, London, S.W. |
| LEACH, J. COMYNS, M.D. Dur., B.Sc. Lond. . | Sturminster Newton. |
| *LEES, E. LEONARD, M.D., C.M. Ed. ... ... | Redland Road, Bristol. |
| LEIGH, W. W. ... ... ... ... ... ... ... ... | Treharris, Glamorganshire. |
| LEWELLYN, JOHN ... ... ... ... ... ... ... | Caerphilly. |
| LIBRARY, BRISTOL MUSEUM AND ... ... ... | Queen's Road, Bristol. |
| LIBRARY, CHELTENHAM ... ... ... ... ... | 5 Royal Crescent, Cheltenham. |
| *LIDDERDALE, JAMES ... ... ... ... ... ... | Henley-on-Thames. |
| *LIGERTWOOD, WALTER... ... ... ... ... ... | University College, Bristol. |
| LILLEY, G. HERBERT, M.D. Brussels ... ... | H.M. Convict Prison, Portland. |
| LIMBERY, THOMAS ... ... ... ... ... ... ... | 184 Commercial Road, Newport, Mon |
| LINDSAY, JAMES A., M.D., M.Ch., M.A. Roy. Univ. I.... ... ... ... ... ... ... ... ... | 37 Victoria Place, Belfast. |

### LIST OF SUBSCRIBERS.

| | |
|---|---|
| LISTER, SIR JOSEPH, Bart., F.R.S. ... ... ... | 12 Park Crescent, London, N.W. |
| LLOYD, WALTER E. ... ... ... ... ... ... ... | 60 York Road, Bristol. |
| *LOWE, T. PAGAN ... ... ... ... ... ... ... | 16 Circus, Bath. |
| LUCKHAM, L. S. ... ... ... ... ... ... ... | 16 Harcourt Terrace, Salisbury. |
| LUCY, REGINALD H., M.B., C.M. Ed. ... ... | 9 Princess Square, Plymouth. |
| | |
| MACDONALD, J. A., M.D., M.Ch. Roy. Univ. I. | 19 East Street, Taunton. |
| MAC DONALD, P. W., M.D., C.M. Aberd. ... | County Asylum, Dorchester. |
| MAC ILWAINE, S. W. ... ... ... ... ... ... ... | South Petherton, Somerset. |
| *MACLEAN, EWEN J., M.D., C.M. Ed. ... ... | Chelsea Hospital for Women, London. |
| MACLEAN, J. CAMPBELL, M.B., C.M Ed. ... | Swindon House, Swindon, Wiltshire. |
| *MACLEOD, H. W. G., M.B., C.M. Ed. ... ... | 23 Victoria Square, Clifton. |
| MACREADY, J. ... ... ... ... ... ... ... ... | 51 Queen Anne Street, London, W. |
| *MADDISON, W. T., M.D. Lond. ... ... ... ... | 172 Stapleton Road, Bristol. |
| MARLEY, HENRY F. ... ... ... ... ... ... | The Nook, Padstow, Cornwall. |
| MARSH, O. E. BULWER ... ... ... ... ... | Parkdale, Newport, Mon. |
| MARSHALL, ARTHUR L., M.B., B.A. Cantab. ... | 56 Rectory Road, London, N. |
| *MARSHALL, HENRY, M.D. Ed., F.R.S.E. ... | 28 Caledonia Place, Clifton. |
| *MARTIN, E. F., M.B. Ed. ... ... ... ... ... | Victoria House, Weston-super-Mare. |
| MARTIN, THEODORE ... ... ... ... ... ... ... | Temple Cloud. |
| MASON, S. B. ... ... ... ... ... ... ... ... ... | 12 Park Terrace, Pontypool. |
| MASON, WILLIAM ... ... ... ... ... ... ... ... | Sedgemoor Cottage, St. Austell. |
| *MATTHEWS, CHARLES E. ... ... ... ... ... | 51 Pembroke Road, Clifton. |
| MAYOR, T. O. ... ... ... ... ... ... ... ... ... | 40 Apsley Road, Clifton. |
| McELFATRICK, JOHN ... ... ... ... ... ... | The Chantry, Mere, Wiltshire. |
| McNICOLL, JOHN ... ... ... ... ... ... ... | High Street, Poole. |
| MEREDITH, JOHN, M.D. Ed. ... ... ... ... | Wellington, Somersetshire. |
| METFORD, J. SEYMOUR ... ... ... ... ... | 34 West Mall, Clifton. |
| *MILLARD, W. J. KELSON, M.D. St. And. ... | Stoke Bishop. |
| MILNE, JOHN, M.B., C.M. Aberd. ... ... ... | Hill Avenue, Bristol. |
| MILWARD, JAMES, M.D. Brussels ... ... ... | 54 Charles Street, Cardiff. |
| *MOLE, HAROLD F. ... ... ... ... ... ... ... ... | 5 Meridian Place, Clifton. |
| MOUAT-BIGGS, C. E. F. ... ... ... ... ... ... | 2 Fauconberg Villas, Cheltenham. |
| MONCKTON, WILLIAM ... ... ... ... ... ... | Portishead. |
| MORGAN, FREDERICK ... ... ... ... ... ... | Uffculme. |
| MORGAN, JOHN ... ... ... ... ... ... ... ... | Langport, Somersetshire. |
| *MORTON, C. A. ... ... ... ... ... ... ... ... | 24 St. Paul's Road, Clifton. |
| *MORGAN, T. WHITWORTH S. ... ... ... ... | Pill. |
| MUNDEN, CHARLES ... ... ... ... ... ... ... | Ilminster. |
| MURRAY, WILLIAM, M.B., C.M. Ed. ... ... | Staple Hill, Gloucestershire. |
| *MYLES, G. T. ... ... ... ... ... ... ... ... ... | 3 Portland Square, Bristol. |
| | |
| NAPIER, T. W. A., M.D., C.M. Aberd. ... ... | Church Street, Egremont, Cheshire. |
| NEALE, B. G. ... ... ... ... ... ... ... ... ... | 34 Waverley Road, Redland. |
| NELL, RICHARD F. ... ... ... ... ... ... ... | 33 Windsor Terrace, Penarth. |
| *NEVILL, W. N., M.D., B.Ch., B.A. Dub. ... | Southville, Bristol. |
| *NEWMAN, CHARLES ... ... ... ... ... ... ... | 156 Cheltenham Road, Bristol. |
| *NEWNHAM, W. H. C., M.B., M.A. Cantab. ... | St. Paul's Road, Clifton |
| NICHOLSON, T. D., M.D., C.M. Ed. ... ... | 2 White Ladies Road, Clifton. |
| NORGATE, R. H. ... ... ... ... ... ... ... | County Asylum, Maidstone. |
| NORMAN, J. H. ... ... ... ... ... ... ... ... | Goodleigh, Winkleigh, Devonshire. |
| *NORTON, J. A., M.D., C.M. Aberd. ... ... ... | 65 Redcliff Hill, Bristol. |
| | |
| ODELL, WILLIAM ... ... ... ... ... ... ... ... | Ferndale, Torquay. |
| *OGILVY, ALEXANDER, M.D., B.Ch., B.A. Dub. | Eye Hospital, Bristol. |
| OPENSHAW, E. HYDE ... ... ... ... ... ... | Moreton Hampstead. |
| *ORMEROD, HENRY ... ... ... ... ... ... ... | Westbury-on-Trym. |

| | |
|---|---|
| Page, G. Shepley ... ... ... ... ... ... ... ... | 78 Old Market Street, Bristol. |
| *Parker, George, M.D., M.A. Cantab. ... ... | 14 Pembroke Road, Clifton. |
| Parry, J. H. ... ... ... ... ... ... ... ... ... | 199 Cheltenham Road, Bristol. |
| Parsons, Francis J. C. ... ... ... ... ... ... | King Square, Bridgwater. |
| Parsons, Frederick ... ... ... ... ... ... ... | Common Hill, Frome. |
| Paterson, Donald Rose, M.D., C.M. Ed.... | 18 Windsor Place, Cardiff. |
| Pauli, G. C. P. ... ... ... ... ... ... ... ... | 240 York Road, Bristol. |
| Peake, G. Arthur ... ... ... ... ... ... ... ... | Rodney Place, Cheltenham. |
| *Penny, F. ... ... ... ... ... ... ... ... ... ... | North Devon Infirmary, Barnstaple. |
| *Penny, W. J. ... ... ... ... ... ... ... ... ... | Richmond Park Road, Clifton. |
| Pentland, Young J. ... ... ... ... ... ... ... | 11 Teviot Place, Edinburgh. |
| Peskett, A. W. C., M.B., B.C., M.A. Cantab. | Burnham, Somersetshire. |
| Phelps, W. Harford G., M.D. Aberd. ... | Beachfield, Weston-super-Mare. |
| Phillips, Edward Picton ... ... ... ... ... ... | High Street, Haverfordwest. |
| Phillips, J. A. ... ... ... ... ... ... ... ... ... | 72 Carlisle Street, Cardiff. |
| *Pickering, C. F. ... ... ... ... ... ... ... ... | 12 Berkeley Square, Bristol. |
| Pippette, Walter... ... ... ... ... ... ... ... | Dawes Road, London, S.W. |
| Plaister, W. H. ... ... ... ... ... ... ... ... | High Road, Tottenham. |
| Pollard, Reginald, M.B. Dur. ... ... ... | Southlands, Torquay. |
| *Pountney, W. E., M.D., C.M. Ed. ... ... | 33a White Ladies Road, Bristol. |
| *Powell, J. J., M.D., Lond. ... ... ... ... | 2 Portland Square, Bristol. |
| Powell, William, M.B. Lond. ... ... ... | Hill Garden, Torquay. |
| Price, R. G., M.D. Dur.... ... ... ... ... ... ... | Carmarthen. |
| Price, William, M.B. Lond. ... ... ... ... | 40 Park Place, Cardiff. |
| *Prichard, Arthur W.... ... ... ... ... ... ... | 6 Rodney Place, Clifton. |
| *Prichard, Augustin, M.D. Berlin ... ... ... | 4 Chesterfield Place, Clifton. |
| *Prichard, J. E., M.B., B.A. Oxon. ... ... | 243 Cheltenham Road, Bristol. |
| *Pring, Arthur, B.A. Cantab. ... ... ... ... | Clyde Road, Bristol. |
| *Prowse, Arthur B., M.D. Lond. ... ... ... | 5 Lansdown Place, Clifton. |

| | |
|---|---|
| Randolph, Charles ... ... ... ... ... ... ... | St. Michael's, Milverton, Somersetshire. |
| *Rattray J. M., M.D., C.M., M.A. Aberd. ... | Frome. |
| Redwood, T. Hall, M.D., C.M., M.A. Dur.... | The Lawn, Rhymney, Monmouthshire. |
| Rendall, John... ... ... ... ... ... ... ... ... | Forestside, Lymington. |
| Rees, Alfred ... ... ... ... ... ... ... ... ... | 46 Loudoun Square, Cardiff. |
| Riddell, Andrew W. ... ... ... ... ... ... ... | St. Mary Bourne. |
| Robathan, G. B. ... ... ... ... ... ... ... ... | The Grove, Risca. |
| Roderick, Sydney J., M.B., C.M. Ed. ... ... | Llanelly. |
| Rodgers, John W., M.B., C.M. Ed. ... ... | Redfield, Gloucestershire. |
| *Rogers, B. M. H., M.D., B.Ch., B.A. Oxon.... | 11 York Place, Clifton. |
| *Ross, R. A. ... ... ... ... ... ... ... ... ... | Lodway Villa, Pill. |
| *Rossiter, George F., M.B. Lond. ... ... | Cairo Lodge, Weston-super-Mare. |
| *Roué, W. Barrett, M.D., M.S. Dur. ... ... | 2 Buckingham Place, Clifton. |
| Row, F. Everard ... ... ... ... ... ... ... ... | 9 Tamar Terrace, Devonport. |
| *Roxburgh, Robert, M.B. Ed. ... ... ... ... | 1 Victoria Buildings, Weston-super-Mare. |
| *Rudge, C. King ... ... ... ... ... ... ... ... | 145 White Ladies Road, Bristol. |
| *Rudge, H. T. ... ... ... ... ... ... ... ... ... | 5 Colston Parade, Bristol. |
| Ruffer, M. Armand, M.D., B.S., M.A. Oxon. | 5 York Terrace, London, N.W. |
| Rutherford, H. T. ... ... ... ... ... ... ... | Park Street, Taunton. |

| | |
|---|---|
| Sandoe, J. W., M.D., B.S. Dur. ... ... ... ... | Broad Clyst. |
| Sawyer, J. A. F. ... ... ... ... ... ... ... ... | Albert Road, Clevedon. |
| Scimonelli, Sigra. Cruce ... ... ... ... ... | Naples. |
| Scott, James, M.B., C.M. Ed.... ... ... ... | 24 Gaolgate Street, Stafford. |
| Scott, Richard J. H. ... ... ... ... ... ... ... | 28 Circus, Bath. |
| Searle, George C. ... ... ... ... ... ... ... | Brixham, Devonshire. |

### LIST OF SUBSCRIBERS.

| | |
|---|---|
| Searle, Richard Burford ... ... ... ... | St. Just. |
| Sebastiano, Bramonti... ... ... ... ... ... ... | 27 Via Balbi, Genoa, Italy. |
| *Semple, H. F.... ... ... ... ... ... ... ... ... | Budleigh Salterton, Devonshire. |
| Seward, W. J., M.B. Lond. ... ... ... ... | Asylum, Colney Hatch, London, N. |
| *Shaw, J. E., M.B., C.M. Ed. ... ... ... ... | 23 Caledonia Place, Clifton. |
| Shaw, Jesse, M.D.... ... ... ... ... ... ... ... | Fort Beaufort, Cape Colony. |
| Sheen, A., M.D. St. And. ... ... ... ... ... | 23 Newport Road, Cardiff. |
| *Sheppard, E. J. ... ... ... ... ... ... ... ... | 11 Richmond Hill, Clifton. |
| *Shorland, Walter E. ... ... ... ... ... | Cecil Road, Bournemouth. |
| Shortridge, Thomas Wood, M.D. Brussels | Honiton. |
| Simons, C. E. G., M.B., C.M. Aberd. ... ... | The Hollies, Merthyr Tydvil. |
| *Sinclair, David, M.B.,C.M. Aberd. ... ... | 6 Whitehall Yard, London, S.W. |
| Sincock, J. Bain ... ... ... ... ... ... ... ... | Friarn Street, Bridgwater. |
| *Skelton, Henry, M.D. St. And. ... ... ... | Downend, Gloucestershire. |
| *Skerritt, E. Markham, M.D., B.S., B.A. Lond. | Richmond Hill, Clifton. |
| Skinner, George H. ... ... ... ... ... ... | Kilmore, Victoria, Australia. |
| *Skinner, Stephen, M.B., C.M. Aberd. ... | Tranent Lawn, Clevedon. |
| *Smith, G. Munro ... ... ... ... ... ... ... | 28 Alma Road, Clifton. |
| *Smith, J. Greig, M.B., C.M., M.A. Ab., F.R.S.E. | 16 Victoria Square, Clifton. |
| Smith, Noble ... ... ... ... ... ... ... ... | 24 Queen Anne Street, London, W. |
| Smith, Rev. Reginald, M.A. Oxon. ... ... | 9 Richmond Hill, Clifton. |
| *Smith, R. Shingleton, M.D., B.Sc. Lond. | Clifton Park, Clifton. |
| Smith, W. Alexander, M.B., M.A. Oxon. ... | Newport, Essex. |
| Snell, Simeon ... ... ... ... ... ... ... ... | 17 Eyre Street, Sheffield. |
| Society, Cardiff Medical (*Hon. Sec.*, Dr. D. R. Paterson). | |
| Society, Plymouth Medical (*Hon. Lib.*, C. E. R. Rendle). | |
| Solly, Reginald V., M.B., B.S. Lond. ... | 7 Dix's Field, Exeter. |
| Solly, S. E. ... ... ... ... ... ... ... ... ... | Colorado Springs, Colorado, U.S.A. |
| Somer, James ... ... ... ... ... ... ... ... ... | Broad Clyst. |
| Soutar, James Greig, M.B., C.M. Ed. ... ... | Barnwood House, Gloucester. |
| *Spencer, W. H., M.D., M.A. Cantab. ... ... | 11 Warrior Square, St. Leonard's-on-Sea. |
| Stamp, W. D. ... ... ... ... ... ... ... ... ... | Ridgeway House, Plympton. |
| Statham, R. Whiteside ... ... ... ... ... ... | Cheddar, Somersetshire. |
| *Steele, Charles, M.D. Dur. ... ... ... ... | 17 Richmond Hill, Clifton. |
| Steele-Perkins, E. B. ... ... ... ... ... ... | 1 Hill's Buildings, Exeter. |
| *Stevens, W. B. ... ... ... ... ... ... ... ... | 16 Gloucester Road, Bristol. |
| *Stevens, W. M., M.D. Lond. ... ... ... ... | 9 Hampton Park, Bristol. |
| Stoddart, F. Wallis ... ... ... ... ... ... ... | 1 Park Street, Bristol. |
| Storry, Frederick W. ... ... ... ... ... ... | Lansdown, Stroud. |
| *Swain, James, M.D., M.S. Lond. ... ... ... | 14 Buckingham Place, Clifton. |
| *Swayne, J. G., M.D. Lond. ... ... ... ... | 74 Pembroke Road, Clifton. |
| *Swayne, S. H.... ... ... ... ... ... ... ... ... | 129 Pembroke Road, Clifton. |
| *Swayne, W. Carless, M.B. Lond.... ... ... | St. Paul's Road, Clifton. |
| Swete, H. Lawton... ... ... ... ... ... ... ... | Fishguard, Pembrokeshire. |
| Symonds, H. P. ... ... ... ... ... ... ... ... | 35 Beaumont Street, Oxford. |
| Symons, John ... ... ... ... ... ... ... ... ... | Buriton House, Penzance. |

| | |
|---|---|
| Tait, Lawson ... ... ... ... ... ... ... ... ... | 7 The Crescent, Birmingham. |
| Taylor, C. Bell, M.D. Edin. ... ... ... ... | 9 Park Row, Nottingham. |
| *Taylor, James... ... ... ... ... ... ... ... ... | 36 Alma Road, Clifton. |
| Taylor, Thomas Henry ... ... ... ... ... | Thornbury, Gloucestershire. |
| Thomas, A. Garrod, M.D., C.M. Ed. ... ... | Clytha Park, Newport, Mon. |
| Thomas, J. Lym ... ... ... ... ... ... ... ... | 28 Charles Street, Cardiff. |
| Thomas, John T. ... ... ... ... ... ... ... ... | Brynhyfryd, Chelston, Torquay. |
| Thomas, W. H.... ... ... ... ... ... ... ... ... | Maesteg, Glamorganshire. |
| Thomson, Eustace B., M.D., C.M. Glasg. ... | Albany Place, Plymouth. |
| Thorne, G. Leworthy, M.D. Aberd. ... ... | Cheriton Fitzpaine. |

*Tivy, W. J. ... ... ... ... ... ... ... ... 8 Lansdown Place, Clifton.
Torbock, W. H., M.D. Pennsylvania ... ... Polruan, Cornwall.
Tosswill, Louis H., M.B., B.A. Cantab. ... 28 West Southernhay, Exeter.
Tribe, Ethel N., M.B. Lond. ... ... ... ... 155 White Ladies Road, Bristol.
Trotter, Leslie B., M.D., C.M. Ed. ... ... Coleford, Gloucestershire.
Turner, A. J. ... ... ... ... ... ... ... ... St. John's Wood Road, Bournemouth.
Tyley, R. P., M.D. St. And. ... ... ... ... Wedmore.

Vachell, Herbert R., M.D., C.M. Aberd.... 18 Newport Road, Cardiff.
Vereker, R. H.... ... ... ... ... ... ... ... Eastleigh, Curry Rivel.
Vinter, Sydney G. ... ... ... ... ... ... Torpoint.

Wade, A. Law, M.D., B.A. Dub. ... ... ... County Asylum, Wells, Somersetshire.
Wade, Charles H., M.A. Oxon. ... ... ... Stanfield, Torquay.
*Waldo, Henry, M.D., C.M. Aberd. ... ... 19 Pembroke Road, Clifton.
*Walker, Cyril H., M.B., B.A. Cantab.... ... St. Paul's Road, Ciflton.
Wallace, Rev. Canon, M.A. Oxon. ... ... 3 Harley Place, Clifton.
*Wallace, John ... ... ... ... ... ... ... 1 Oriel Terrace, Weston-super-Mare.
Walsh, Leslie H. ... ... ... ... ... ... ... Royal United Hospital, Bath.
Walter, William Henry, M.D. Brussels ... Winslow.
Ward, J. L. W. ... ... ... ... ... ... ... Victoria Street, Merthyr Tydvil.
*Wathen, J. Hancocke ... ... ... ... ... 16 York Place, Clifton.
Watson, J. Adam ... ... ... ... ... ... ... 39 Dennington Park, London, N.W.
Watters, George T. B., M.D., C.M. Ed. ... Stonehouse, Gloucestershire.
Waugh, Alexander ... ... ... ... ... ... Midsomer Norton.
Weatherly, Lionel A., M.D., C.M. Aberd. Bailbrook House, Bath.
Webber, William W.... ... ... ... ... ... Crewkerne.
Webster, Norman P. ... ... ... ... ... ... George Place, Guernsey.
*Webster, Thomas ... ... ... ... ... ... Redland Hill, Bristol.
*Wedmore, C. Ernest, M.B., M.A. Cantab. Stoke Bishop.
Wethered, Frank J., M.D. Lond. ... ... 34 Queen Anne Street, London, W.
*Whicher, A. H. ... ... ... ... ... ... ... Midsomer Norton.
Whitfeld, W. Clarke ... ... ... ... ... Baggally Street, Hereford.
Whyte, Andrew, M.D., C.M. Aberd. ... ... Honddû House, Brecon.
Wickham, W. ... ... ... ... ... ... ... ... Hill House, Tetbury.
Wigan, C. A., M.D. Dur. ... ... ... ... ... Portishead.
Wigmore, J., M.D. Roy. Univ. I. ... ... ... Twerton-on-Avon.
Wilcox, Henry, M.B. Aberd. ... ... ... ... Fleet, Hampshire.
*Wilding, James, M.D. Dur. ... ... ... ... Sydenham Road, Bristol.
Willcox, R. Lewis ... ... ... ... ... ... Warminster.
Willett, George G. D. ... ... ... ... ... Keynsham, Somersetshire.
William, John, M.D. St. And. ... ... ... ... Brynmeurig, Bethesda.
Williams, Charles ... ... ... ... ... ... 116 Derby Road, Bootle, Liverpool.
Williams, D. J. ... ... ... ... ... ... ... Greenfield House, Llanelly.
Williams, H. Egerton ... ... ... ... ... The Mount, Newport, Mon.
*Williams, Lionel H. ... ... ... ... ... ... Thornbury, Gloucestershire.
*Williams, P. Watson, M.D. Lond. ... ... 2 Lansdown Place, Clifton.
Williams, Peter ... ... ... ... ... ... ... Ferryside, Carmarthenshire.
*Windsor-Aubrey, H. W. ... ... ... ... ... Coronation Road, Bristol.
Winterbotham, L. ... ... ... ... ... ... Bays Hill, Cheltenham.
*Wintle, Colston ... ... ... ... ... ... ... Children's Hospital, Bristol.
Woodford, E. Russell, M.D., C.M. Ed. ... 4 Prospect Terrace, Ventnor.
Woodhead, G. Sims, M.D., C.M. Ed. ... ... 1 Nightingale Lane, London, S.W.
Woollcombe, Walter L. ... ... ... ... ... 16 Lockyer Street, Plymouth.

Yelf, Leonard K., M.D. St. And. ... ... ... Moreton-in-Marsh.

### Exchange-List.

### AMERICA.

### ARGENTINE REPUBLIC.

*Monthly.*

*Anales del Circulo Médico Argentino.* Buenos Ayres: Mariano Moreno.

### CANADA.

*Monthly.*

*The Canadian Practitioner.* Toronto: The J. E. Bryant Company.

*The Montreal Medical Journal.* Montreal: Gazette Printing Company.

### MEXICO.

*Twice a month.*

*Gaceta Médica.* Mexico: Avenida 2 Oriente, 726.

### PERU.

*Monthly.*

*La Crónica Médica.* Lima: 24 Calle de Bodegones.

### UNITED STATES.

*Weekly.*

*Medical Record.* New York: William Wood & Co.

*Medical Review.* St. Louis: 914 Locust Street.

*The Cincinnati Lancet-Clinic.* Cincinnati: 199 West 7th Street.

*The Journal of the American Medical Association.* Chicago: 68 Wabash Avenue.

*The Medical and Surgical Reporter.* Philadelphia: 147-149-151 North Tenth Street.

*The Medical News.* Philadelphia: Lea Brothers & Co.

*The New York Medical Journal.* New York: D. Appleton & Co.

*Fortnightly.*

*The American Practitioner & News.* Louisville: John P. Morton and Company.

*Twice a month.*

*The Medical Age.* Detroit: George S. Davis.

*Monthly.*

*Buffalo Medical and Surgical Journal.* Buffalo: *Times* Printing House.

*Bulletin of the Johns Hopkins Hospital.* Baltimore: The Johns Hopkins Press.

*Index Medicus.* Detroit: George S. Davis.

*International Medical Magazine.* Philadelphia: International Medical Magazine Company.

*Journal of Cutaneous and Genito-Urinary Diseases.* New York: D. Appleton & Co.

*Notes on New Remedies.* New York: Lehn & Fink.

*The American Journal of Obstetrics & Diseases of Women and Children.* New York: William Wood & Co.

*The American Journal of Ophthalmology* St. Louis: J. H. Chambers & Co.

*The American Journal of the Medical Sciences.* Philadelphia: Lea Brothers & Co.

*The Journal of Nervous and Mental Disease.* New York: 25 West 45th Street.

*The Post-Graduate.* New York: 226 East 20th Street.

*The Saint Louis Medical and Surgical Journal.* Saint Louis: The Commercial Printing Company.

*The Therapeutic Gazette.* Detroit: George S. Davis.

*The Universal Medical Journal.* Philadelphia: The F. A. Davis Company.

*Quarterly.*

*Mathews' Medical Quarterly.* Louisville: John P. Morton & Company.

*The Alienist and Neurologist.* St. Louis: Ev. E. Carreras.

*Yearly.*

*Annual of the Universal Medical Sciences.* Philadelphia: The F. A. Davis Company.

*Transactions of the American Surgical Association.* Philadelphia: William J. Dornan.

*Transactions of the New York Academy of Medicine.* New York: Stettiner, Lambert & Co.

### ASIA.

### CHINA.

*Quarterly.*

*The China Medical Missionary Journal.* Shanghai: The American Presbyterian Mission Press.

### INDIA.

*Monthly.*

*The Indian Medico - Chirurgical Review.* Bombay: Rao & Co.

### JAPAN.

*Monthly.*

*The Sei-I-Kwai Medical Journal.* Tokio 6 Shin Sakana Chō, Kyōbashi Ku.

### AUSTRALASIA.

### AUSTRALIA.

*Monthly.*

*The Australasian Medical Gazette.* Sydney: L. Bruck.

*The Australian Medical Journal.* Melbourne: Stillwell & Co.

### NEW ZEALAND.

*Quarterly.*

*The New Zealand Medical Journal.* Dunedin: J. Wilkie and Co.

### EUROPE

### AUSTRIA.

*Weekly.*

*Wiener Klinische Wochenschrift.* Vienna: Wilhelm Braumüller.

### BELGIUM.

*Monthly.*

*Archives de médecine et de chirurgie pratiques.* Brussels: C. Duculot-Roulin.

*Bulletin de l'Académie royale de médecine de Belgique.* Brussels: F. Hayez.

### BRITISH ISLES.

*Weekly.*

*British Medical Journal.* London: 429 Strand.

*Medical Press and Circular.* London: A. A. Tindall.

*The Clinical Journal.* London: E. Knight.

*The Hospital.* London: 428 Strand.

*The Lancet.* London: 423 Strand.

*The Sanitary Record.* London: Newspaper Distributing Agency.

*Monthly.*

*The Birmingham Medical Review.* Birmingham: Hall & English.

*The British Journal of Dermatology.* London: H. K. Lewis.

*The Journal of Laryngology, Rhinology, and Otology.* London: F. J. Rebman.

*The Medical Chronicle.* Manchester: John Heywood.
*The Medical Magazine.* London: Southwood, Smith, & Co.
*The Practitioner.* London: Macmillan & Co.
*Edinburgh Medical Journal.* Edinburgh: Oliver and Boyd.
*The Glasgow Medical Journal.* Glasgow: Alex. Macdougall.
*The Dublin Journal of Medical Science.* Dublin: Fannin & Co.

*Quarterly.*

*Archives of Surgery.* London: J. & A. Churchill.
*The Asclepiad.* London: Longmans, Green, & Co.
*The British Gynæcological Journal.* London: John Bale & Sons.
*The International Journal of Microscopy & Natural Science.* London: Baillière, Tindall & Cox.
*The Quarterly Medical Journal.* Sheffield: Pawson & Brailsford.

*Half-yearly.*

*The Liverpool Medico-Chirurgical Journal.* Liverpool: Medical Institution.
*The Retrospect of Medicine.* London: Simpkin & Co. Limited.

*Yearly.*

*Clinical Society's Transactions.* London: Longmans, Green & Co.
*Guy's Hospital Reports.* London: J. & A. Churchill.
*Medico-Chirurgical Transactions.* London: Longmans, Green & Co.
*St. Bartholomew's Hospital Reports.* London: Smith, Elder, & Co.
*St. Thomas's Hospital Reports.* London: J. & A. Churchill.
*The Medical Annual.* Bristol: John Wright & Co.
*The Year-Book of Treatment.* London: Cassell & Company, Limited.
*Transactions of the Royal Academy of Medicine in Ireland.* Dublin: Fannin & Co.
*Year-Book of Scientific and Learned Societies.* London: C. Griffin & Co.

*Occasionally.*

*The Directory of Second-hand Booksellers.* Rochdale: James Clegg.
*Transactions of the West London Medico-Chirurgical Society.* London: Baillière, Tindall & Cox.

DENMARK.

*Weekly.*

*Hospitalstidende.* Copenhagen: Jacob Lund.

FRANCE.

*Three times a week.*

*Gazette des hôpitaux.* Paris: 4 Rue de l'Odéon.
*L'Union médicale.* Paris: Octave Doin.

*Weekly.*

*Bulletin de l'Académie de médecine.* Paris: G. Masson.
*Gazette des hôpitaux de Toulouse.* Toulouse: 3 Avenue Lafayette.
*La France médicale.* Paris: 14 Rue des Pyramides.
*Le Progrès médical.* Paris: 14 Rue des Carmes.

*Twice a month.*

*Gazette de Gynécologie.* Paris: 12 Rue de Châteaudun.
*Revue internationale de Bibliographie médicale.* Paris: 23 Rue Racine.

*Monthly.*

*Archives cliniques de Bordeaux.* Bordeaux: Feret et Fils.
*Archives de Neurologie.* Paris: 14 Rue des Carmes.
*Revue de Laryngologie, d'Otologie et de Rhinologie.* Paris: Octave Doin.
*Revue générale d'Ophtalmologie.* Paris: G. Masson.

*Bi-monthly.*

*Nouvelle Iconographie de la Salpêtrière.* Paris: L. Battaille et Cie.

GERMANY.

*Weekly.*

*Centralblatt für Chirurgie.* Leipzig: Breitkopf & Härtel.

*Centralblatt für innere Medicin.* Leipzig: Breitkopf & Härtel.

*Münchener medicinische Wochenschrift.* Munich: J. F. Lehmann.

*Monthly.*

*Fortschritte der Krankenpflege.* Berlin: H. Kornfeld.

GREECE.

*Weekly.*

Γαληνὸς. Athens: N. G. Passare.

HOLLAND.

*Weekly.*

*Nederlandsch Tijdschrift voor Geneeskunde.* Amsterdam: F. van Rossen.

ITALY.

*Weekly.*

*Gazzetta Medica Lombarda.* Milan: 33 Via Spiga.

*Monthly.*

*Annali di Ostetricia e Ginecologia.* Milan: Lodovico Felice Cogliati.

NORWAY.

*Monthly.*

*Norsk Magazin for Lægevidenskaben.* Christiania: Det Steenske Bogtrykkeri.

PORTUGAL.

*Weekly.*

*A Medicina Contemporanea.* Lisbon: José Antonio Rodrigues.

ROUMANIA.

*Twice a month.*

*Spitalul.* Bucharest: Thoma Basilescu.

RUSSIA.

*Weekly.*

*Medycyna.* Warsaw: 80 Allée de Jerusalem.
*Vrach.* St. Petersburg: C. Ricker.
*St. Petersburger Medicinische Wochenschrift.* St. Petersburg: Carl Ricker.

*Monthly.*

*Finska Läkaresällskapets Handlingar.* Helsingfors: Helsingfors Central-Tryckeri.

SPAIN.

*Weekly.*

*El Siglo Médico.* Madrid: Magdalena, 36.

*Twice a month.*

*Revista de Ciencias Médicas.* Barcelona: 9 Calle Fortuny.

SWEDEN.

*Quarterly.*

*Nordiskt Medicinskt Arkiv.* Stockholm: P. A. Norstedt & Söner.

TURKEY.

*Twice a month.*

*Gazette Médicale d'Orient.* Constantinople: A. Christidis.

### PUBLICATIONS RECEIVED.

From JOHN BALE & SONS, London:
*Chorea.* By OCTAVIUS STURGES, M.D. Second Edition. 1893.
*Rodent Cancer.* By J. INGLIS PARSONS, M.D. 1893.
*Trephining in its Ancient and Modern Aspects.* By JOHN FLETCHER HORNE, M.D. 1894.
*Congenital Affections of the Heart.* By GEORGE CARPENTER, M.D. 1894.

From THOMAS BEST BURROW, London:
*The News.* March 9, 1894.

From CASSELL & COMPANY, LIMITED, London:
*The Rectum and Anus.* By CHARLES B. BALL. Second Edition. 1894.

From THE CHURCH DEFENCE INSTITUTION, London:
*The National Church.* Jan.—March, 1894.

From J. & A. CHURCHILL, London:
*Death from Traumatic Fever.* By JOHN D. MALCOLM, M.B. 1893.
*The Open-Air Treatment of Phthisis.* By W. BEZLY THORNE, M.D. 1894

From WILLIAM F. CLAY, Edinburgh:
*Gynæcological Diagnosis.* By N. T. BREWIS, M.B. 1894.

From T. & A. CONSTABLE, Edinburgh:
*Atlas of Clinical Medicine.* By BYROM BRAMWELL, M.D. Vol. II., Part III. 1893.

From W. A. DUNKERLEY, London:
*To-Day.* Jan. 13, 1894.

From PAUL DUPONT, Paris:
*Revue des Associations professionnelles.* Dec. 25, 1893.

From EYRE & SPOTTISWOODE, London:
*The Art of Living in Australia.* By PHILIP E. MUSKETT. [1893.]

From WM. F. FELL & CO., Philadelphia:
*Modern Homeopathy.* By WILLIAM W. BROWNING, M.D. [Second Edition.] 1894.

From GOVERNMENT PRINTING OFFICE, Washington:
*Index-Catalogue of the Library of the Surgeon-General's Office.* Vol. XIV. Sutures—Universally. 1893.

From CHARLES GRIFFIN & COMPANY, LIMITED, London:
*An Introduction to Midwifery.* By ARCHIBALD DONALD, M.D. 1894.

From THE GROCERY WORLD PUBLICATION CO., Philadelphia:
*Grocery World and Fruit Trade Bulletin.* Dec. 23, 1893.

From H. HELBING, London:
*Helbing's Pharmacological Record.* Nos. XVII., XXIV., XXV. May—Dec., 1893; Jan., 1894.

From THE "JAPAN HERALD" OFFICE, :
*Description of a Newly-devised Sanitary Building.* By W. VAN DER HEYDEN, M.D. 1893.

From THE JOHNS HOPKINS PRESS, Baltimore:
*The Johns Hopkins Hospital Reports.* Vol. III., Nos. 7-8-9. *Report in Gynecology.* II. 1894.

From H. K. LEWIS, London:
*Baths in Skin Diseases.* By LESLIE PHILLIPS, M.D. 1893.
*Antiseptics in Midwifery.* By ROBERT BOXALL, M.D. 1894.
*Headache and other Morbid Cephalic Sensations.* By HARRY CAMPBELL, M.B. 1894.
*Infectious Diseases.* By LOUIS C. PARKES, M.D. 1894.
*Common Neuroses.* By JAMES FREDERIC GOODHART, M.D. Second Edition. 1894.
*Dwelling Houses.* By W. H. CORFIELD, M.D. Third Edition. 1894.
*Sprains.* By C. W. MANSELL MOULLIN, M.D. Second Edition. 1894.

